# Optical Coherence Tomography Angiography (OCT-A) in a Patient with Occult Retinal Dysfunction

**DOI:** 10.1155/2019/4349692

**Published:** 2019-06-24

**Authors:** Juliana Wons, Jana Dinges, Matthias D. Becker, Stephan Michels

**Affiliations:** ^1^City Hospital Triemli, Zurich, Switzerland; ^2^University of Heidelberg, Germany; ^3^University of Zurich, Switzerland

## Abstract

Multimodal imaging techniques were performed in a patient with a newly emerged visual field defect; a missing retinal lesion on fundus examination made the diagnosis challenging but infrared imaging showed a larger area of retinal abnormality temporal to the fovea. Indocyanine green angiography (IA) showed late hypofluorescence and there was mild hyperautofluorescence which is known from acute zonal occult outer retinopathy (AZOOR). Despite normal fluorescein angiography (FA) results, a perfusion loss in the outer retinal layer was detected by OCT-A. Similar OCT-A findings were recently described in patients with acute macular neuroretinopathy (AMN).* Methods*. The methods included FA and IA, spectral domain optical coherence tomography (SD-OCT), near infrared imaging, and autofluorescence imaging (AF), as well as OCT-A.* Patient. *A 36-year-old patient who suffered from acute symptoms of photopsia and scotoma on her left eye. She had an influenza*-*like illness two weeks earlier. The scotoma could be verified by visual field testing.* Results*. The affected retinal zone showed mild fading of external limiting membrane (ELM) and a disorganisation of the ellipsoid zone (EZ) on SD-OCT. OCT-A revealed a large area of reduced perfusion in the outer retinal vascular layer.* Conclusion*. OCT-A can help to detect reduced capillary network in patients with visual field defects and no visible fundus changes. This case seems to have features of different occult retinal disorders such as AZOOR and AMN.

## 1. Introduction

AZOOR was initially described by Gass in 1992 as an outer retinal dysfunction that affects young Caucasian patients, more often women than men [1, 2]. Photopsia and visual field defects are the main symptoms [2]. The dysfunction can be uni- or bilateral [2]. Some patients suffer from an initial episode of flu like symptoms. Only slight or no fundus changes are detectable [3].

Until now it remains unclear if the retinal lesions progress due to an autoimmune process, idiopathic, or due to a viral affection [2]. In some cases a mild periphlebitis in the affected area is described [3]. After the initial phase, sometimes mild fundus changes like pigment shift or a slight chorioretinal scaring as well as a bonespicule-like pigmentation can be observed [2]. OCT images show often initially fading of the ellipsoid zone (EZ), potentially due to higher reflectivity of the outer nuclear layer (ONL), and show loss of photoreceptor outer segments during the course of disease [3]. Fluorescein angiography is usually normal at the onset of a lesion. Over time with degenerative changes at the level of the RPE, an early FA hyperfluorescence can be detected.

AMN was initially described by Bos and Deutman in 1975 [4]. They described cases of patients with red and wedge-shaped paracentral lesions [4]. The apices of these lesions often point towards the fovea. Patients suffer from acute paracentral scotoma; visual acuity is mostly normal or mildly reduced. The symptoms can occur uni- or bilateral. While fundus examination often shows only mild or sometimes even no fundal changes, the lesions are well visualised by red-free or infrared imaging, typically as dark or grey regions with well-demarcated margins [5]. Patients are more often young and female; a number of supposed triggers have been reported, such as flu-like illness, fever, oral contraceptives, and some sympathomimetics [5]. The defect remains stable or can occasionally recover [5].

Before standard use of OCT in ophthalmic diagnostics, AMN was rarely diagnosed. OCT imaging shows wedge shaped hyperreflectivity in the outer nuclear layer or a fading EZ in patients with AMN [6]. Studies using OCT-angiography have revealed an ischemia in the deep retinal plexus as a pathogenic mechanism [7, 8].

## 2. Case Report

A 36-year-old female had photopsia on her left eye for ten days. Furthermore, she noticed a dark spot on the nasal side of the left eye. Two weeks earlier, she had suffered from influenza-like symptoms. There was no history of previous general or eye disease, neither operations nor treatments.

## 3. Results

**Figure 1:**
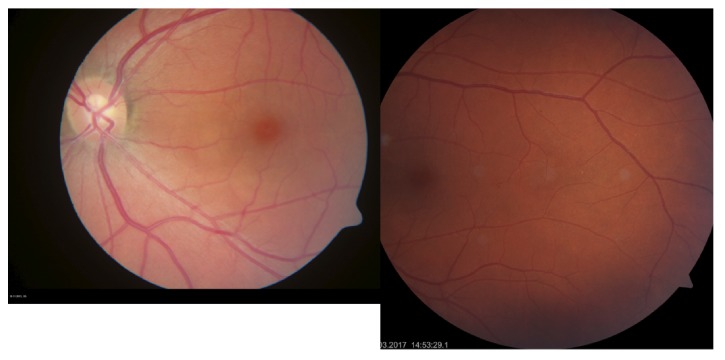
Color fundus photography of the left eye.

**Figure 2:**
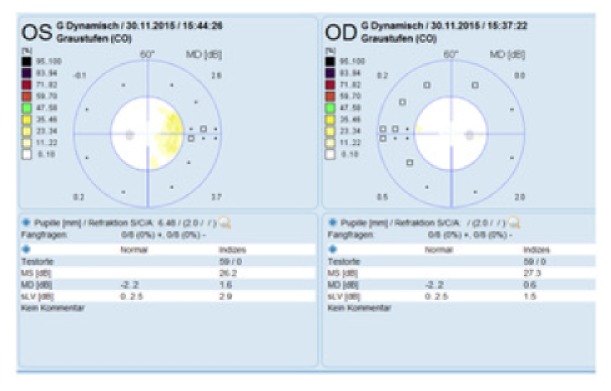
Octopus perimetry confirms the nasal defect on the left eye.

**Figure 3:**
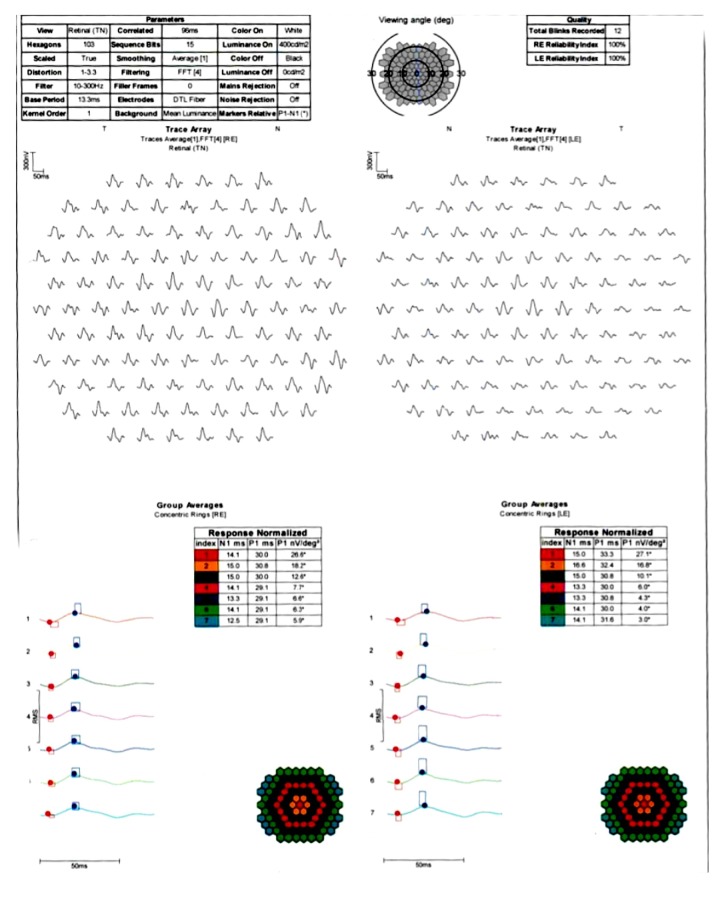
Multifocal ERG with reduced response signal in the area of the temporal retina on the left eye.

**Figure 4:**
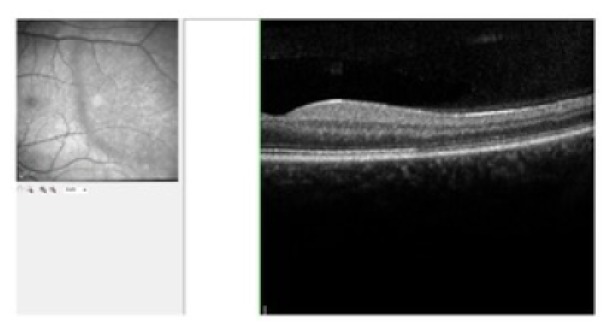
Optical coherence tomography showing fading of the ellipsoid zone and infrared imaging indicating affected zone.

**Figure 5:**
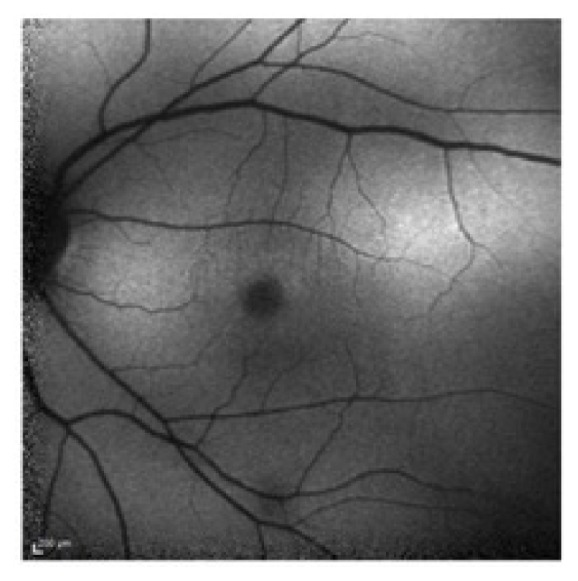
Autofluorescence image of the left eye.

**Figure 6:**
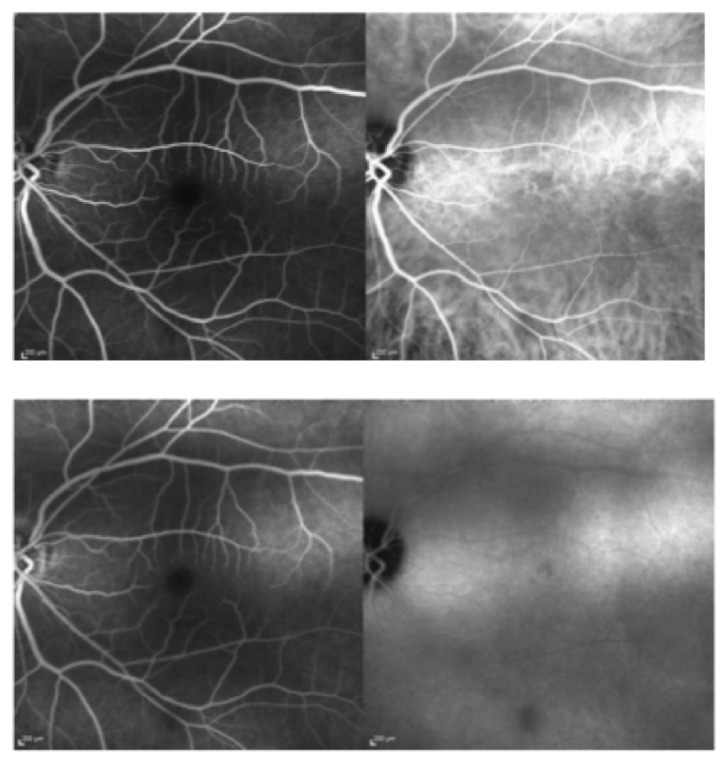
On the left early and late fluorescein (FA), and on the right early and late indocyanine green (IA) angiography. Late IA reveals a hypofluorescent area temporal to the fovea.

**Figure 7:**
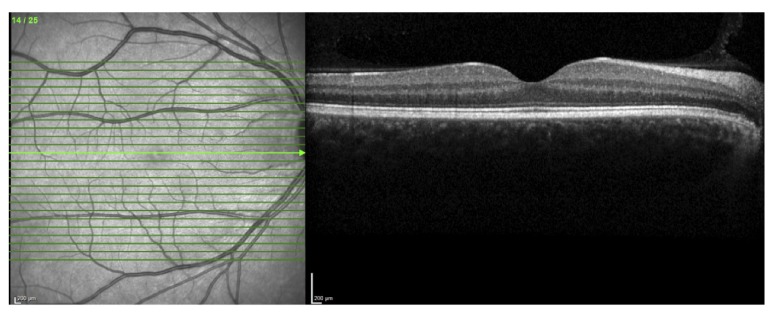
Optical coherence tomography and infrared imaging of the right eye.

The BCVA was 20/20 on both eyes, normal eye pressure, and no signs of inflammation in the anterior chamber. There was no vitreous haze or vitreous destruction. The fundus examination showed no obvious pathology (Figure 1). The scotoma was confirmed by using 30° perimetry (Figure 2). Blood tests for uveitis were negative, and screening for rheumatic diseases showed normal findings. An electroretinogram revealed reduced receptor potentials in the impaired retinal zone on the left eye corresponding to the visual defect (Figure 3). The near infrared image showed a significant demarcation line temporal to the fovea, and there was a fading of the EZ on the SD-OCT (Spectralis®; Heidelberg Engineering GmbH, Heidelberg, Germany) image (Figure 4). The autofluorescence (AF) imaging (Figure 5) showed very mild focal hyperautofluorescence superotemporally to the fovea. FA revealed normal retinal perfusion, there was no sign of ischemia, no leakage, nor hyperfluorescence. However, ICG revealed an area of hypofluorescence temporal to the fovea (Figure 6). This region correlates to the scotoma on the left eye. No pathologic changes were found in the right eye (Figure 7.).

**Figure 8:**
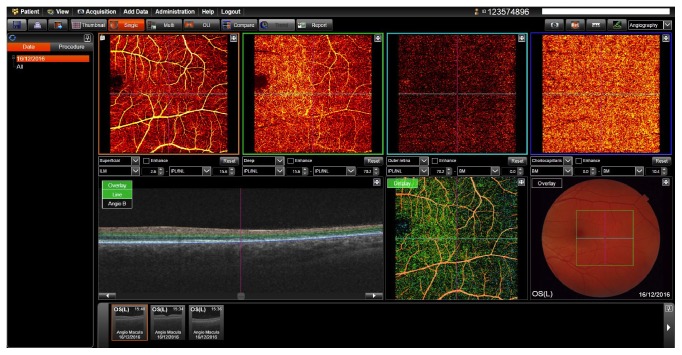
Optical coherence tomography angiography (OCTA) shows a rarefication of the deep retinal capillaries. Superficial retinal capillaries and choroidal circulation do not seem to be affected.

OCT angiography (OCT-A) was performed using the Topcon, DRI OCT Triton/Swept source OCT-A. The affected area of the left eye was demarcated and changes in the density of the retinal capillaries were demonstrated (Figure 8). These changes were only in the deep retinal vascular layer.

## 4. Discussion

Comparable findings in OCT-A were recently described in several cases of patients with AMN [7–9]. But only one article mentioned changes on OCT-A in AZOOR. Pichi et al. describe reduced deep capillary flow on OCT-A in an inactive peripapillary lesion of a patient with AZOOR [10]. In our case, the first OCT-A was performed several months after the first visit, the lesion was inactive at that time and our findings would therefore be conclusive with the other article.

There are several findings that would fit to the diagnosis of AZOOR, such as hypofluorescence in late phase of ICG, thinning of the outer plexiform layer, outer nuclear layer and disruption of ellipsoid zone. Fluorescein angiography is usually normal at the onset of a lesion, with subsequent degenerative changes at the level of the RPE. AZOOR shows loss of photoreceptor outer segments, beginning with abnormalities in the ellipsoid line. In the course of disease early FA hyperfluorescence from depigmentation of the RPE produce a window defect [11]. We followed the patient over more than 2 years and the lesion did not change neither in SD-OCT, nor in FA.

Often there are no changes on infrared imaging in AZOOR. This patient has changes on near infrared, a feature that is often a hallmark of AMN. The ellipsoid line and outer segments are affected in AMN and also the deep retinal capillaries. Most AZOOR patients show affection of the peripapillary zone and only rarely does it spare the area around the disc but affect an area temporal to the macula. The multifocal ERG can show reduced amplitudes both in AZOOR and in AMN.

Some features in this case are not typical for the diagnosis of AMN either. There were no classical wedge shaped lesions pointing towards the macula and no hyperreflective changes in the SD-OCT. However, the demarcated area in the near infrared image and the ischemia in the outer retinal layer are consistent with AMN [6, 9].

In appearance of newer imaging techniques making diagnosis by OCT and OCT-A is in discussion. Some authors claim that there might be the risk of determining diseases on a fragile basis where technical artefacts may lead to false interpretations. Otherwise, the modern imaging methods may help us to improve our current classification of retinal diseases.

The pathology of the AZOOR complex is still under debate. Anti-retinal bodies could play, according to a recent publication, a key role leading to the damage of the retinal area in AZOOR [12]. A dysfunction in the blood-retina barrier due to inflammation or autoimmune process could be involved in the pathogenic process [12]. The current challenge, as discussed by Mrejen et al., is the lack of consistency regarding the definition of the disease and the clinical findings [11]. An advantage in the challenge of finding the diagnosis of this relatively rare disease can be achieved through modern multimodal imaging techniques.

## 5. Conclusion

OCT-A identifies retinal ischemic diseases on clinically undetectable fundus lesions.

The changes in the perfusion of the deep retinal capillaries indicated by OCT-A in this young female patient with visual field defect helped to establish the diagnosis of an outer retinal dysfunction, combining features of AZOOR and AMN. A better understanding of the pathophysiology in these rare occult retinal diseases may be achieved by increasing use of OCT-A.
